# Modulation of Neural Activity in the Temporoparietal Junction with Transcranial Direct Current Stimulation Changes the Role of Beliefs in Moral Judgment

**DOI:** 10.3389/fnhum.2015.00659

**Published:** 2015-12-14

**Authors:** Hang Ye, Shu Chen, Daqiang Huang, Haoli Zheng, Yongmin Jia, Jun Luo

**Affiliations:** ^1^College of Economics, Interdisciplinary Center for Social Sciences at Zhejiang UniversityHangzhou, China; ^2^School of Economics and International Trade, Zhejiang University of Finance and EconomicsHangzhou, China; ^3^Neuro and Behavior EconLab, Zhejiang University of Finance and EconomicsHangzhou, China

**Keywords:** theory of mind, moral judgment, beliefs and outcomes, temporoparietal junction, transcranial direct current stimulation

## Abstract

Judgments about whether an action is morally right or wrong typically depend on our capacity to infer the actor’s beliefs and the outcomes of the action. Prior neuroimaging studies have found that mental state (e.g., beliefs, intentions) attribution for moral judgment involves a complex neural network that includes the temporoparietal junction (TPJ). However, neuroimaging studies cannot demonstrate a direct causal relationship between the activity of this brain region and mental state attribution for moral judgment. In the current study, we used transcranial direct current stimulation (tDCS) to transiently alter neural activity in the TPJ. The participants were randomly assigned to one of three stimulation treatments (right anodal/left cathodal tDCS, left anodal/right cathodal tDCS, or sham stimulation). Each participant was required to complete two similar tasks of moral judgment before receiving tDCS and after receiving tDCS. We studied whether tDCS to the TPJ altered mental state attribution for moral judgment. The results indicated that restraining the activity of the right temporoparietal junction (RTPJ) or the left the temporoparietal junction (LTPJ) decreased the role of beliefs in moral judgments and led to an increase in the dependance of the participants’ moral judgments on the action’s consequences. We also found that the participants exhibited reduced reaction times both in the cases of intentional harms and attempted harms after receiving right cathodal/left anodal tDCS to the TPJ. These findings inform and extend the current neural models of moral judgment and moral development in typically developing people and in individuals with neurodevelopmental disorders such as autism.

## Introduction

In everyday life, a harm caused by an action is morally worse than an equivalent harm caused by omission, and a harm intended as the means to a goal is morally worse than an equivalent harm foreseen as the side effect of a goal (Cushman et al., 2006; Young and Koenigs, 2007). Moral judgment entails judging others’ actions on the dimension of right and wrong, but this requires not only the outcomes of these actions but also the cognitive ability to think about another person’s beliefs and intentions, which is known as “theory of mind” (Young and Saxe, 2008).

A number of recent studies indeed demonstrate that mental state information (e.g., desire, belief, intention) is one of the crucial inputs into moral decision-making (for a review, see Young and Tsoi, 2013). Evidence from developmental psychology also shows that children (even preverbal infants) start condemning negative intent that does not result in negative outcome (see Baird and Astington, 2004; Killen et al., 2011). But when beliefs and outcomes are incongruent with each other, there are different ways that this incongruence can behaviorally present itself relying on the valence of the conflicting belief and outcome (Patil and Silani, 2014).

Cushman (2008) found that judgments of punishment depended jointly on mental states and the causal relationship of an agent to a harmful consequence. An account of these phenomena has been proposed that distinguished two processes of moral judgment (Young et al., 2007; Cushman et al., 2013; Cushman, 2013): one which begins with harmful outcome and attributes condemnation to the causally responsible agent, and the other which begins with an action and analyses the mental states responsible for that action.

Neuroimaging studies have investigated the selectivity and domain specificity of these brain regions for thinking about another person’s thoughts. These regions, which comprise the “theory of mind network, ” include the medial prefrontal cortex (MPFC), precuneus (PC), right superior temporal sulcus (RSTS), and bilateral temporal-parietal junction (TPJ; Gallagher et al., 2000; Vogeley et al., 2001; Ruby and Decety, 2003; Saxe and Kanwisher, 2003; Aichhorn et al., 2009).

The precise role of these brain regions in theory of mind for moral judgment has been the topic of recent researches (Young et al., 2007; Young and Saxe, 2008). Specifically, the TPJ exhibits increased activity whenever participants read about a person’s beliefs in nonmoral (Saxe and Kanwisher, 2003; Saxe and Powell, 2006) or moral contexts (Young et al., 2007, 2010b). However, fMRI cannot demonstrate direct causal relationships between the activities in these brain regions and mental state attribution for moral judgment.

Noninvasive brain stimulation techniques, such as rTMS, allow for the study of the decision consequences of externally restrained brain activity in healthy participants and thus the establishment of causal connections between the brain and decisions without many of the confounds inherent to natural lesion studies (Rafal, 2001; Robertson et al., 2003). Young et al. (2010a) and Jeurissen et al. (2014) used rTMS to transiently suppress activity in the right temporoparietal junction (RTPJ) and provided evidence for the causal role of this structure in mental state attribution for moral judgment.

Transcranial direct current stimulation (tDCS) has some advantages relative to rTMS because it induces a stronger modulatory effect on brain activity (Nitsche and Paulus, 2000; Romero et al., 2002), allowing for reliable sham stimulation (Gandiga et al., 2006). Importantly, anodal tDCS increases excitability in targeted brain regions, which can transiently enhance decisions and judgment in healthy humans (Fregni et al., 2005; Wassermann and Grafman, 2005).

The goal of the present study was to alter moral judgments by modulating the cortical excitability over the TPJ in healthy adults. To measure the participants’ capacities to infer the actor’s mental state attributions in moral judgment, we presented the participants with moral scenarios in which (i) the protagonist acts on either a negative belief (e.g., that he or she will cause harm to another person) or on a neutral belief and (ii) the protagonist either causes a negative outcome (e.g., harm to another person) or a neutral outcome (Young et al., 2007; Young and Saxe, 2008). Participants made judgments on a scale of 1 (permissible) to 10 (forbidden), which were regarded as their condemnation ratings towards the behaviors described.

Previous findings have provided direct evidence supporting the critical role of the RTPJ in mediating belief attribution for moral judgment, For example Young et al. (2010a) revealed that the disruption of the RTPJ with TMS led participants to rely their judgments less on the actor’s mental states, and Sellaro et al. (2015) found that participants who received anodal tDCS over the RTPJ assigned less blame to accidental harms compared to participants who received sham stimulation. However, a direct causal relationship between left temporoparietal junction (LTPJ) and mental state attribution for moral judgments has not been studied. In the present study, we sought to firstly test whether modulating the activity of the LTPJ activity with tDCS would also influence the role of beliefs on moral judgments. Therefore, we performed an experiment to investigate whether bilateral stimulation of the TPJ (anodal stimulation of the right and cathodal stimulation of the left TPJ or vice versa) would alter mental state attribution for moral judgments. Our findings suggested that restraining the RTPJ or LTPJ with tDCS decreased the role of beliefs in moral judgment. Combining our findings with those of previous work, we infer that the RTPJ and LTPJ commonly represent the ability to use mental states in moral judgment and that both are responsible for the role of belief in moral judgment.

Besides the difference in stimulation electrode positions from previous evidence, the present study has novel assignment for moral judgment task and classification for story context. The previous experiments demonstrated the role of the RTPJ on belief attribution by comparing participants’ moral judgments following TMS to the RTPJ and TMS to a control brain region (Young et al., 2010a), or investigating participants’ performance on the moral judgment task before and after having received anodal, cathodal, or sham tDCS over the RTPJ (Sellaro et al., 2015). These studies selected and randomly distributed moral stories among different treatments (including active stimulations and sham stimulation) and different tasks (pre-tDCS and post-tDCS task) to test their hypotheses. However, they haven’t made sure the balance and similarity of moral stories across the treatments and tasks. In this study, each participant was required to complete a similar (and we demonstrated the similarity) moral judgment task before and after receiving tDCS. Therefore, we combined within-subject and between-subject design in this experiment to test the causal role of the bilateral TPJ regarding mental states in moral judgment.

In addition, how one should act toward another depends on whether the target is a friend, a stranger, a subordinate, or an authority (Dungan and Young, 2012). Therefore, we have assigned two different types of story context that involved economic interests and relationships with friends in moral judgment task to explore the role of TPJ on the actors’ mental state attributions for moral judgment across different contexts. Analyses indicated that in conditions of neutral belief, the condemnation ratings of contexts involving economic interests were lower than those of contexts involving relationship with friends. Moreover, in conditions of negative belief with contexts involving economic interests, the condemnation ratings were lower after receiving right anodal/left cathodal tDCS. These findings indicate that the restraining effect of tDCS on the LTPJ in the role of beliefs in moral judgment depends on moral context.

## Materials and Methods

### Subjects

We recruited 54 healthy college students (32 females; mean age 22.11 years, ranging from 19–30 years) to participate in our experiment. All participants were right-handed and naïve to tDCS and moral judgment tasks, and they had no history of psychiatric illness or neurological disorders. The participants were randomly assigned to receive right anodal/left cathodal tDCS over TPJ (*n* = 18, 11 females), left anodal/right cathodal tDCS over TPJ (*n* = 18, 11 females) or sham stimulation over TPJ (*n* = 18, 10 females). Each participant received 50 RMB yuan (approximately 7.995 US dollars) for their participation. Participants gave written informed consent before entering the study, which was approved by the Zhejiang University ethics committee. No participants reported any adverse side effects about pain on the scalp or headaches after the experiment.

### Transcranial Direct Current Stimulation (tDCS)

tDCS was induced by two saline-soaked surface sponge electrodes (35 cm^2^). Direct current was constant and delivered by a battery-driven stimulator (Multichannel noninvasive wireless tDCS neurostimulator, Starlab, Barcelona, Spain), which was controlled through a Bluetooth signal. It was adjusted to induce cortical excitability of the target area without causing any physiological damage to the participants. Various orientations of the current had various effects on the cortical excitability. Generally speaking, anodal stimulation enhances cortical excitability, whereas cathodal stimulation inhibits it (Nitsche and Paulus, 2000).

**Figure 1:**
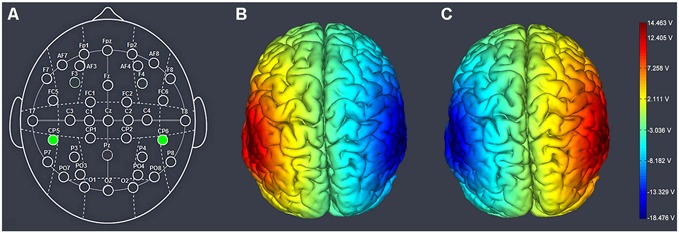
**Electrode placements. (A)** Schematic of the electrode positions based on the EEG 10–20 system. **(B)** Left anodal/right cathodal stimulation over the temporoparietal junction (TPJ) of the human brain. **(C)** Right anodal/left cathodal stimulation over the TPJ of the human brain. The axis represents the range of input voltage from −18.476 to 14.463 V.

TPJ was localized with location CP5 (left) and CP6 (right) on an EEG cap laid out according to the International 10–20 System (**Figure 1A**). Participants were randomly assigned to one of the three single-blinded stimulation treatments. For right anodal/left cathodal stimulation, the anodal electrode was placed over the CP6 according to the international EEG 10–20 system, while the cathodal electrode was placed over the CP5. For left anodal/right cathodal stimulation the placement was reversed. The anodal electrode was placed over CP5 and the cathodal electrode was placed over CP6 (**Figures 1B,C**). Therefore, the target electrode (either the anode or the cathode) was centered over CP6/CP5; the return electrode was placed over CP5/CP6. The reason we chose a bifrontal electrode montage was to provide stimulation able to enhance the activity of one side of the TPJ while simultaneously diminish the other side. For sham stimulation, the procedures were totally the same but the current lasted only for the first 30 s. The participants may have felt the initial itching, but actually there was no current for the rest of the stimulation. This method of sham stimulation has been shown to be reliable (Gandiga et al., 2006). The current had an intensity of 2 mA with 15 s of ramp up and down, the safety and efficiency of which was shown in previous studies.

**Figure 2:**
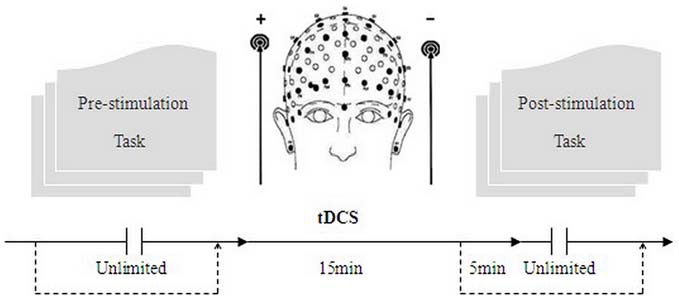
**Schematic representation of the experimental process.** The participant was required to perform the first moral task before stimulation. After 15 min of stimulation, each participant was asked to complete the second task while the stimulation was continued for another 5 min.

After the participant finished the first moral judgment task (the computer program for these tasks was written in visual C#) which was similar to Young’s design (Young et al., 2010a), the laboratory assistant put a tDCS device on his/her head for stimulation and removed him/her from the computer screen. After 15 min of stimulation, the participant was then asked to complete the latter moral judgment task with the stimulation being delivered for another 5 min (**Figure 2**).

### Task and Procedure

**Figure 3:**
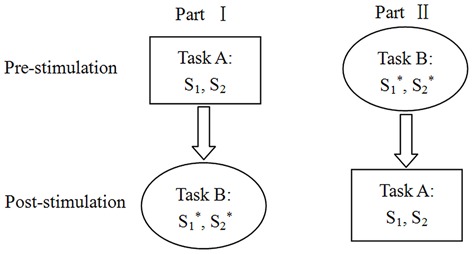
**Experimental design.** The half of participants (Part I) were required to complete the moral judgment task A (S_1_, S_2_) before stimulation and complete the moral judgment task B (S*_1_, S*_2_) after stimulation, while the rest of participants (Part II) were required to complete task B (S*_1_, S*_2_) before stimulation and complete task A (S_1_, S_2_) after stimulation.

The experiment included two moral judgment tasks. Each participant was required to complete a moral judgment task before receiving tDCS and to complete another moral judgment task after receiving tDCS. To eliminate the sequence effect of the two tasks, we randomly assigned half of the participants (Part I) to complete moral judgment task A (including story S_1_ and S_2_) before receiving tDCS and to complete moral judgment task B (including story S*_1_ and S*_2_) after receiving tDCS; the remaining participants (Part II) completed task B before receiving tDCS and completed task A after receiving tDCS (**Figure 3**). Each story was based on a type of context that involved economic interests (S_1_ and S*_1_) or relationships with friends (S_2_ and S*_2_).

There were four conditions in each story that included belief (negative vs. neutral) and outcome (negative vs. neutral) factors to yield a 2 × 2 design. Specifically, they were intentional harm (negative belief and negative outcome), accidental harm (neutral belief and negative outcome), attempted harm (negative belief and neutral outcome) and nonharm (neutral belief and neutral outcome). Stories were presented in cumulative segments, each presented for 8 s, describing in a fixed order: (i) background; (ii) foreshadow; (iii) belief; and (iv) action. The background was identical across conditions. Stories were then removed from the screen and replaced with a question about the moral permissibility of the action. Participants made judgments on a scale of 1 (permissible) to 10 (forbidden) using a computer keyboard, which were regarded as their condemnation ratings towards the behaviors described. The time limit for responding was 6 s. The reaction times were recorded and all of the participants had made judgments within the time limit.

**Figure 4:**
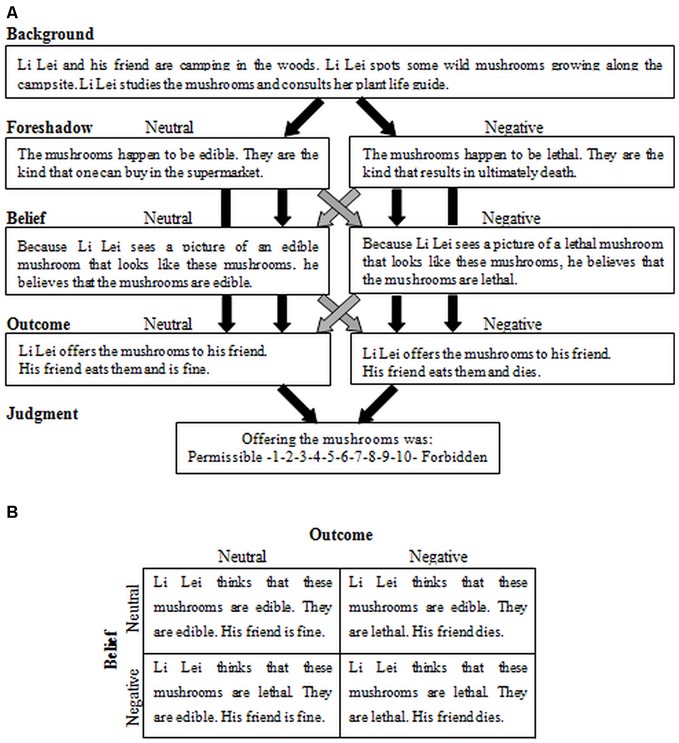
**Task design and experimental stimuli. (A)** Schematic representation of sample scenario. Light-colored arrows mark the combinations of “Foreshadow” and “Belief” for which the belief is false. “Foreshadow” information foreshadows whether the action will result in a neutral or negative outcome. “Belief” information states whether the protagonist holds a belief that he is in a neutral situation and that action will result in a neutral outcome (neutral belief) or a belief that he is a negative situation and that action (or inaction) will result in a negative outcome (negative belief). **(B)** Combination of belief (neutral vs. negative) and outcome (neutral vs. negative) factors yielded a 2 × 2 design with four conditions.

The participants were required to read and make judgments about two moral stories with four conditions respectively before receiving tDCS. After completing this moral judgment task, they had a break and received tDCS for 15 min. Subsequently, they were required to read and make judgments about another two stories with four conditions respectively while receiving stimulation for another 5 min. The latter moral judgment task was similar to the first moral judgment task to avoid learning effects in the within-subject design experiment. Both tasks included two stories (S_1_ and S*_1_; S_2_ and S*_2_) with four conditions (**Figure 4**). The same participant saw all four variations of the same story in both sessions, eight stories pre-stimulation and eight-stories post-stimulation, for a total of 16 stories. On average each story consisted of about 91 words, and the number of words was matched across conditions and tasks. When the subjects completed the two moral judgment tasks, they were asked to complete a questionnaire before finally receiving their payment.

### Data Analysis

We first tested the similarity of tasks A and B using repeated measures analyses of variance (ANOVA). Giving the two tasks were equivalent in terms of condemnation ratings and reaction times before receiving tDCS, it ensured us to compare the performance of the participants before and after receiving tDCS. Then we used repeated measures ANOVA to test if the stimulation had changed the participants’ moral judgment in different conditions, including condemnation ratings and reaction times. As we distinguished between the contexts that involved economic interests and relationships with friends, all these tests were applied firstly without consideration of the difference between the two contexts (the pooled sample) and then treating context as a within-subjects factor (sample with context). The statistical analyses were performed using SPSS statistical software (SPSS Inc., Chicago, IL, USA).

## Results

### The Pooled Sample

**Figure 5:**
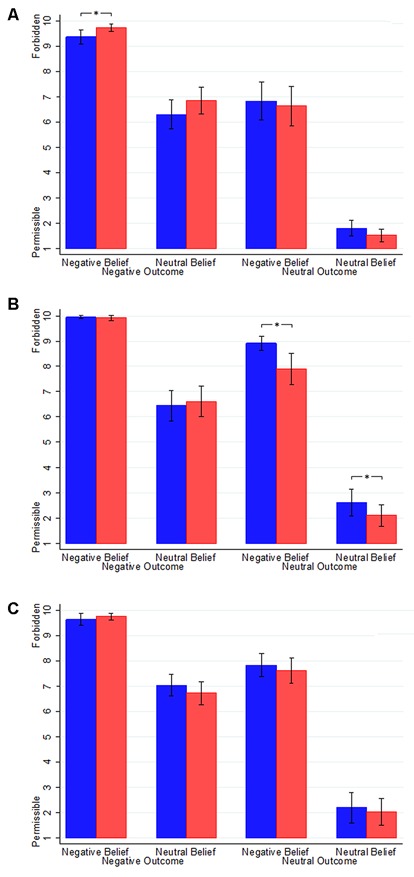
**Data of condemnation ratings. (A)** The condemnation ratings of participants with four conditions in the right anodal/left cathodal treatment before and after stimulation. **(B)** The condemnation ratings of participants with four conditions in the left anodal/right cathodal treatment before and after stimulation. **(C)** The condemnation ratings of participants with four conditions in the sham treatment before and after stimulation. Blue columns, pre-transcranial direct current stimulation (tDCS); red columns, post-tDCS. Error bars indicate 95% confidence intervals. Asterisks indicate statistical significance of difference within-subject.

The mean condemnation ratings and standard deviation information of different conditions and different stimulation types are shown in **Figure 5** and **Table 1**. We first tested whether task A was different from task B before receiving tDCS using repeated measures ANOVA with Belief (neutral vs. negative) and Outcome (neutral vs. negative) as within-subjects factors and Task (A vs. B) as a between-subjects factor. There was significant effect of task neither in condemnation ratings [*F*_(1,106)_ = 0.007, *P* = 0.931] nor in reaction times [*F*_(1,106)_ = 0.752, *P* = 0.388], which made it reasonable to regard the two tasks as equivalent and compare the performance of the participants before and after receiving the stimulations. Meanwhile, we found significant effect of Belief [*F*_(1,106)_ = 671.932, *P* < 0.001], Outcome [*F*_(1,106)_ = 419.632, *P* < 0.001] and a significant interaction of Belief and Outcome [*F*_(1,106)_ = 109.063, *P* < 0.001] in condemnation ratings.

**Table 1:** **The mean condemnation ratings and SD across conditions and stimulation types**.

Condition	R Anodal/L Cathodal		L Anodal/R Cathodal		Sham	
	Before	After	Before	After	Before	After
Intentional harm	9.36 (0.99)	9.72 (0.51)	9.94 (0.23)	9.92 (0.37)	9.64 (0.83)	9.75 (0.5)
Accidental harm	6.31 (2.01)	6.86 (1.90)	6.44 (2.12)	6.11 (2.16)	7.03 (1.52)	6.72 (1.61)
Attempted harm	6.83 (2.70)	6.64 (2.75)	8.92 (0.97)	7.89 (2.23)	7.83 (1.61)	7.61 (1.82)
Nonharm	1.81 (1.06)	1.53 (0.88)	2.61 (1.89)	2.11 (1.53)	2.19 (2.15)	2.03 (1.9)

Since there was no significant difference between condemnation ratings and reaction times for the two moral judgment tasks, the difference before and after the stimulations could be attributed to the effect of tDCS. We ran a repeated measures ANOVA with Belief (neutral vs. negative), Outcome (neutral vs. negative) and Time (before vs. after tDCS) as within-subjects factors and stimulation type (right anodal/left cathodal, left anodal/right cathodal or sham) as a between-subjects factor. Significant effects of Belief [*F*_(1,105)_ = 845.032, *P* < 0.001] and Outcome [*F*_(1,105)_ = 586.439, *P* < 0.001] were observed, which meant that the participants’ condemnation ratings of moral judgment in conditions of negative belief (mean = 8.671) were higher than that of neutral belief (mean = 4.354). Similarly, conditions of negative outcome (mean = 8.192) were more condemned than conditions of neutral outcome (mean = 4.833). Moreover, the interaction of Belief and Outcome also had a significant effect [*F*_(1,105)_ = 4.454, *P* = 0.014]. *Post hoc* analysis using bonferroni corrections indicated that conditions of intentional harm (mean = 8.755) and attempted harm (mean = 8.588) were less permissible than both conditions of accidental harm (mean = 4.398) and nonharm (mean = 4.310). We also found significant effect of stimulation type [*F*_(2,105)_ = 5.289, *P* = 0.006].

Importantly, we found a slightly significant three-way interaction involving Outcome, Time and stimulation type [*F*_(2,105)_ = 3.185, *P* = 0.045]. Analysis showed that in conditions of negative outcome, participants rated higher in condemnation after receiving right anodal/left cathodal tDCS [before: mean = 7.833; after: mean = 8.292; *P* = 0.005], especially towards intentional harm [*P* = 0.001]. On the other hand, in conditions of neutral outcome, participants rated lower in condemnation after receiving left anodal/right cathodal tDCS [before: mean = 5.764; after: mean = 5.000; *P* < 0.001], both towards attempted harm [*P* = 0.001] and nonharm [*P* = 0.015]. These findings might indicate that restraining the activity of the RTPJ/LTPJ decreased the role of beliefs in moral judgments and led to the participants’ moral judgments being more dependent on the actions’ consequences.

We paid attention to reaction time as well. Applying the above repeated measures ANOVA, we found a significant effect of Time [*F*_(1,105)_ = 7.571, *P* = 0.007]. It is easy to understand that the reaction times after stimulation were shorter than before because that the participants were more familiar with the task. Moreover, the three-way interaction of Belief, Time and stimulation type was trending towards significant [*F*_(2,105)_ = 2.749, *P* = 0.069]. *Post hoc* analysis indicated that the reaction times in conditions of negative belief were significantly shorter after left anodal/right cathodal tDCS [*P* = 0.004], while in conditions of neutral belief the reaction times were significantly shorter after sham stimulation [*P* = 0.023]. The mean reaction time and standard deviation information are displayed in supplementary materials.

Lastly, we checked whether the sequence of the two tasks would influence the participants’ moral judgment. Repeated measures ANOVAs showed no significant effect of sequence in condemnation ratings [*F*_(1,102)_ = 0.154, *P* = 0.695] or in reaction times [*F*_(1,102)_ = 1.633, *P* = 0.204].

### Sample with Context

To test the effect of context, we added Context (economic interests vs. relationships with friends) as a within-subjects factor into the repeated measures ANOVAs in section “The Pooled Sample”. We first tested the similarity of tasks A and B. No significant effect of Task in condemnation ratings [*F*_(1,52)_ = 0.004, *P* = 0.947] or in reaction times [*F*_(1,52)_ = 0.407, *P* = 0.526] was observed. Apart from the significant effects of Belief [*F*_(1,52)_ = 427.022, *P* < 0.001], Outcome [*F*_(1,52)_ = 254.778, *P* < 0.001] and a significant interaction of Belief and Outcome [*F*_(1,52)_ = 65.701, *P* < 0.001] in condemnation ratings as in sections “The Pooled Sample”, there was also a significant interaction of Context and Belief [*F*_(1,52)_ = 7.379, *P* = 0.009]. Analysis indicated that in conditions of neutral belief, the condemnation ratings of contexts involving economic interests were lower than those of contexts involving relationships with friends [*P* = 0.010].

We then performed repeated measures ANOVA with Context, Belief, Outcome and Time as within-subjects factors and stimulation type as a between-subjects factor. Again we found significant effects of Belief [*F*_(1,51)_ = 473.717, *P* < 0.001] and Outcome [*F*_(1,51)_ = 321.762, *P* < 0.001], which meant that the participants’ ratings of moral judgment in conditions of negative belief were higher than that of neutral belief, as well as conditions of negative outcome were more condemned than conditions of neutral outcome. The interaction of Belief and Outcome also had a significant effect [*F*_(1,51)_ = 65.255, *P* < 0.001]. In addition, we found significant effects of Context [*F*_(1,51)_ = 5.391, *P* = 0.024], which meant that contexts involving economic interests [mean = 6.419] was less condemned than those of contexts involving relationships with friends [mean = 6.606]. Besides, there was a significant four-way interaction involving Context, Belief, Time and stimulation type [*F*_(2,51)_ = 3.871, *P* = 0.027]. Analysis indicated that in conditions of negative belief with contexts involving economic interests, the condemnation ratings were lower after receiving right anodal/left cathodal tDCS [*p* = 0.014]. There was also a similar but slightly less significant effect in conditions of negative belief with contexts involving economic interests [*P* = 0.069].

As for the reaction time, we found a significant effect of Time [*F*_(1,51)_ = 4.517, *P* = 0.038] similar to section “The Pooled Sample”. A significant four-way interaction of Context, Belief, Outcome and stimulation type was also observed [*F*_(2,51)_ = 3.908, *P* = 0.026], indicating that in conditions of accidental harm, the reaction times of contexts involving economic interests were longer than those of contexts involving relationships with friends in sham stimulation [*P* = 0.013]. The mean reaction time and standard deviation information are displayed in supplementary materials. At last, no significant effect of sequence was observed in condemnation ratings [*F*_(1,48)_ = 0.083, *P* = 0.774] or in reaction times [*F*_(1,48)_ = 0.855, *P* = 0.360].

## Discussion

Human moral judgment often represents a response that depends on various factors and features that include not only the agent’s beliefs but also the agent’s desires (Cushman, 2008), their consequences (Greene et al., 2001), the agent’s prior record (Kliemann et al., 2008), the cause that leads to harm (Cushman et al., 2008), whether the action was coerced by external circumstances (Woolfolk et al., 2006; Krebs et al., 2014), (etc., Valdesolo and DeSteno, 2006; Young et al., 2010a). In the present study, we manipulated two of these factors, the agent’s belief and the outcomes of the action, and tested whether the effect of modulating activity in the TPJ with tDCS was specific to the agent’s mental state attribution for moral judgment.

This study corroborated and complemented the previous finding by Young et al. (2010a), which postulated that disrupting RTPJ function reduces the influence of beliefs on moral judgment. We found that restraining the RTPJ via tDCS caused the participants to judge attempted harms and nonharm as less morally forbidden and more morally permissible, while restraining the LTPJ via tDCS caused the participants to judge accidental harms and intentional harms as more morally forbidden and less morally permissible. Thus, suppressing the activity in the RTPJ or LTPJ disrupted the capacity to use mental states in moral judgment.

To verify the robustness of our results, we modified a related experimental design based on that of Young et al. (2010a). Previous neurostimulation experiments of human decision-making have primarily utilized between-subject design (Knoch et al., 2006; Fecteau et al., 2007a,b; Boggio et al., 2010; Young et al., 2010a). However, the corresponding results lack statistical power due to the heterogeneity of the participants, especially when the samples are small. Our experiment adopted a within-subject design to avoid this interference from the heterogeneity of the participants. Provided that the multiple exposures are independent, this design makes it possible for causal estimates to be obtained by examining how individual decisions change after receiving stimulation.

Furthermore, the previous studies haven’t made sure the balance of moral stories across the treatments. In this study, each participant was required to complete similar moral judgment task before and after receiving tDCS (active stimulations and sham stimulation). We also demonstrated that task A was equivalent to task B before receiving tDCS either in terms of condemnation ratings or reaction times, which made it reasonable to compare the performance of the participants before and after receiving the stimulations. Since there was no significant difference between the two moral judgment tasks, the difference before and after the stimulations could be attributed to the effect of tDCS.

Generally, moral judgments are robust to different demographic factors such as gender, age, ethnicity, and religion, but many complexities in moral judgment are still left unresolved. No comprehensive model or taxonomy of moral judgment thus far has accounted for its full diversity. Some models call for a division of the moral space based on the content, and there is work going one on about the role of intentions as a function of the moral content (Shweder et al., 1997; Rozin et al., 1999; Dungan and Young, 2012). This content-based approach also proves fruitful in explaining different emotional responses to different kinds of moral violations. Specifically, there is evidence that individuals have made difference for moral judgment between stranger and friend (Ma, 1989; Smetana et al., 2006; Kurzban et al., 2012).

To consider the context effect on both participants’ condemnation ratings and the effects of tDCS for TPJ, we have assigned two different types of moral context that involved friend relationships (harm to her/his friend) and economic interests (harm to her/his customer)—as food-safety problems in China have contributed to a rapid decline of social trust (Yan, 2012)—as stories of moral judgment and separately tested whether the modulation of activity in the TPJ with tDCS changed the agents’ mental state attributions for moral judgment in both the friend relationship and economic interest contexts. Analyses indicated that in conditions of neutral belief, the condemnation ratings of contexts involving economic interests were lower than those of contexts involving relationship with friends. Moreover, in conditions of negative belief with contexts involving economic interests, the condemnation ratings were lower after receiving right anodal/left cathodal tDCS. These findings indicate that the restraining effect of tDCS on the LTPJ in the role of beliefs in moral judgment depends on moral context.

The present study also investigated the participants’ reaction times for moral judgments and found that the participants who received restraint of the RTPJ exhibited reduced reaction times in both the cases of intentional harms and attempted harms when the story involved economic interests. Because restraining the RTPJ significantly decreased the capacity to infer the actor’s intentions in moral judgment, the participants could easily make judgments that primarily considered the attribution of action’s consequence when the role of belief in moral judgment was reduced.

Many studies have shown that both the RTPJ and the LTPJ play essential roles in the theory of mind and that the activities of these two brain regions are associated with the understanding of social intentions (Ciaramidaro et al., 2007; Sommer et al., 2007; Aichhorn et al., 2009; Centelles et al., 2011). Recent fMRI studies have also suggested that the bilateral TPJ are recruited for the encoding and integrating process of beliefs (Young and Saxe, 2008). Specifically, Young et al. (2010a) used TMS to the RTPJ to disrupt the capacity to integrate belief information. Samson et al. (2004) reported evidence from brain-damaged patients that indicated that the patients with lesions in the LPTJ region exhibit impairment in false belief tasks.

In the present study, we also found that restraining the RTPJ or LTPJ via tDCS decreased the role of beliefs in moral judgment. Combining our findings with those of previous work, we infer that the RTPJ and LTPJ commonly represent the capacity to use mental states in moral judgment and that both are responsible for the role of belief in moral judgment. After receiving tDCS to restrain the activities of the RTPJ or LTPJ, the role of beliefs in moral judgment is reduced. In the four conditions of moral stories, the participants placed more weight on the attribution of the action’s consequences but not on intentions in moral judgment. Specifically, after restraining the activity of the TPJ, participants judged intentional harms and accidental harms as more morally forbidden and less morally permissible, and the participants judged attempted harms and nonharm as less morally forbidden and more morally permissible. These effects might also depend on stories’ context of moral judgment.

In conclusion, our findings provide important information about the effects of tDCS on mental states in moral judgment. These findings might be helpful for the study and treatment of neurodevelopmental disorders, such as autism spectrum disorders (ASDs). Children with ASDs are unable to impute beliefs to others (Baron-Cohen et al., 1985). Even high functioning adults with ASDs have a persistent impairment in spontaneous mentalizing (i.e., the automatic ability to attribute mental states to the self and others; Senju et al., 2009). Furthermore, the impairment in the processing of the mental states of others in autism is associated with reduced RTPJ activity (Kana et al., 2009). Therefore, we believe that this study might inform neural models of moral judgment and moral development in typically developing people and in individuals with neurodevelopmental disorders such as autism (Koster-Hale et al., 2013).

Additionally, both folk moral judgments and legal decisions depend on agent’s ability to make judgment for the consequences of an individual’s actions to the beliefs and intentions of actions. Our experiments revealed that the mental state attribution of moral judgment, especially in cases involving attempted harm and accidental harm, depends critically on neural activity in the TPJ. Future studies should explore the relevance of these findings for the real-life judgments made by judges and juries who routinely make very detailed distinctions based on mental state information.

Since the same participant saw all four variations of the same story during the experiment, we acknowledged this design may increase demand characteristics for the task as participants could figure out the differences of four conditions. However, we aimed to study whether tDCS to the TPJ (active stimulation treatments) altered mental state attribution for moral judgment. Therefore, the possibility of those demand characteristics which were perceived by the participants would not lead to biased experimental results. In addition, it was noted that the robustness of the current findings across diverse moral contexts remained to be determined because of the limited number of stimuli used in the experiment.

Another limitation of the present study is that we were unable to determine whether the effect on mental state attribution of moral judgment was solely attributable to the modulation of the activity in the RTPJ or whether the changes in moral judgment resulted from altering the balance of activity across the bilateral TPJ. With regard to the tDCS polarity effects, Jacobson et al. (2012) conducted a meta-analytical review aimed to investigate the homogeneity/heterogeneity of the effect sizes of the anodal-excitation and cathodal-inhibition effects dichotomy in both motor and cognitive functions. They found that the anode electrode is applied over a cognitive area, in most cases, it will cause an excitation as measured by a relevant cognitive task. However, the cathodal-inhibition effects seems to be robust only in the motor and sensory cortex but there is wide variation for cognitive studies. Therefore, our finding that the influence of modulating activity in the bilateral TPJ with tDCS on the role of beliefs in moral judgment, to a large extent, may resulted from anodal-excitation effects, rather than cathodal-inhibition effects. Future experiments may include neuroimaging measures to explore the neural changes associated with the neuromodulation that lead to decision-making effects and also to explore other paradigms of stimulation, such as unilateral stimulation.

## Author Contributions

HY, SC, DH, HZ, YJ and JL designed experiment; DH, HZ, JL performed experiment; SC analyzed data; HY drew figures; SC, DH, HZ and JL wrote the manuscript.

## Conflict of Interest Statement

The authors declare that the research was conducted in the absence of any commercial or financial relationships that could be construed as a potential conflict of interest.
