# Supplementary material for: Modulation of Neural Activity in the Temporoparietal Junction with Transcranial Direct Current Stimulation Changes the Role of Beliefs in Moral Judgment
Source: Front Hum Neurosci. 2015 Dec 14;9:659. doi: 10.3389/fnhum.2015.00659 (PMC4677104; doi:10.3389/fnhum.2015.00659)
Supplement: Supplementary file 1 [file Table_1.docx]

***Supplementary Material***

**Modulation of neural activity in** **the temporoparietal junction with transcranial direct current stimulation changes the role of beliefs in moral judgment**

**Hang Ye^1^, Shu Chen^1^, Daqiang Huang^1^, Haoli Zheng^1^, Yongmin Jia^1^, Jun Luo^2^***

^1^College of Economics, Interdisciplinary Center for Social Sciences at Zhejiang University, Hangzhou, China

^2^School of Economics and International Trade, Zhejiang University of Finance and Economics, Hangzhou, China

*** Correspondence:** Jun Luo, School of Economics and International Trade, Zhejiang University of Finance and Economics, No. 18, Xueyuan Street, Hangzhou, 310018, China.

luojun_zju@hotmail.com

**Content**

Scenarios and Stories.............................................................................................................................................2

The Mean Condemnation Ratings and SD across Conditions and Stimulation Types of Sample with Contexts.................................................................................................................................................................4

The Mean Reaction Time and SD across Conditions and Stimulation Types of the Pooled Sample...................5

The Mean Reaction Time and SD across Conditions and Stimulation Types of Sample with Contexts..............6

**Scenarios**

Scenarios are organized in the following order:

1. Background: information to set the scene (identical across all conditions)

2. Foreshadow: information indicating the outcome (neutral, negative)

3. Belief: the protagonist’s belief about the situation (neutral, negative)

4. Action: the protagonist’s action and its outcome (neutral, negative)

**S_1_: Mushrooms.**

Li Lei and his friend are camping in the woods. Li Lei spots some wild mushrooms growing along the campsite. Li Lei studies the mushrooms and consults her plant life guide.

The mushrooms happen to be edible. They are the kind that one can buy in the supermarket and put in salad.

The mushrooms happen to be lethal. They are the kind that results in painful convulsions and ultimately death.

Li Lei sees a picture of an edible mushroom in his book that looks just like these mushrooms at the campsite, so he believes that the mushrooms are edible.

Li Lei sees a picture of a lethal mushroom in his book that looks just like these mushrooms at the campsite, so he believes that the mushrooms are lethal.

Li Lei offers the mushrooms to his friend. His friend eats them and is fine.

Li Lei offers the mushrooms to his friend. His friend eats them and dies.

Offering the mushrooms was:

**S_1_^*^: Driving.**

Yang Fei and his friend are driving on a mountain road. They almost can’t see the way because of thick smog. They get lost. Yang Fei gets off alone and tries to find the way.

There is a flat area ahead. Driving along this road is safe and finally leads to highway.

There is a cliff ahead. Driving along this road is dangerous and ultimately results in death.

Yang Fei walks along this road for a moment. Yang Fei thinks there is a flat area ahead, so he believes that driving along this road is safe to his friend.

Yang Fei walks along this road for a moment. Yang Fei thinks there is a cliff ahead, so he believes that driving along this road will lead to his friend’s death.

Yang Fei lets his friend drive along this road by himself. His friend drives along this road and is fine.

Yang Fei lets his friend drive along this road by himself. His friend drives along this road and dies.

Directing the way was:

**S_2_: Pesticides.**

Luo Fang is a vegetable vendor. She wants to use a kind of pesticide in the planting to improve productivity. Luo Fang studies the pesticide and searches related knowledge in the internet.

The pesticide does not cause harm to humans. It is often used on large scale irrigation by farmers.

The pesticide can cause harm to humans. It is prohibited for using on irrigation by local government.

Luo Fang searches some knowledge about the pesticide and infers that chemical compositions of this pesticide are safe, so she believes that the pesticide does not cause harm to humans.

Luo Fang searches some knowledge about the pesticide and infers that chemical compositions of this pesticide are poisonous, so she believes that the pesticide can cause people to die.

Luo Fang uses this pesticide on vegetable irrigation. Customers eat her vegetables and are fine.

Luo Fang uses this pesticide on vegetable irrigation. Customers eat her vegetables and die.

Using the pesticide was:

**S_2_^*^: Preservatives.**

Lin Yan is a meat supplier. She finds some preservatives in the corner of factory. She wants to use these preservatives in the production process to increase profits.

The preservatives are not harmful to humans. They are common preservatives which can prevent meat from going bad.

The preservatives are harmful to humans. They are no longer used for corrosion protection, because it is illegal.

Li Yan asks a food safety expert some information about the preservatives. The expert tells her the preservatives are safe, so she believes that the preservatives does not cause harm to humans.

Li Yan asks a food safety expert some information about the preservatives. The expert tells her the preservatives are very poisonous, so she believes that the preservatives are lethal to humans.

Lin Yan uses the preservatives in the production process. Customers eat her meat and are fine.

Lin Yan uses the preservatives in the production process. Customers eat her meat and die.

**The Mean Condemnation Ratings and SD across Conditions and Stimulation Types of Sample with Contexts**

| Context | Condition | R Anodal/L Cathodal | | L Anodal/R Cathodal | | Sham | |
| --- | --- | --- | --- | --- | --- | --- | --- |
|  |  | Before | After | Before | After | Before | After |
| economic interests | Intentional harm | 9.11 (1.13) | 9.67 (0.59) | 9.94 (0.24) | 9.89 (0.47) | 9.72 (0.67) | 9.72 (0.57) |
|  | Accidental harm | 6.11 (2.00) | 6.44 (2.09) | 6.06 (1.89) | 6.11 (2.27) | 6.78 (1.56) | 6.61 (1.91) |
|  | Attempted harm | 6.94 (2.84) | 7.06 (2.78) | 9.00 (0.91) | 7.83 (2.41) | 8.11 (1.53) | 7.72 (1.81) |
|  | Nonharm | 1.83 (1.15) | 1.39 (0.78) | 2.06 (1.51) | 1.83 (1.10) | 2.11 (2.14) | 2.00 (2.03) |
| relationships with friends | Intentional harm | 9.61 (0.78) | 9.78 (0.43) | 9.94 (0.24) | 9.94 (0.24) | 9.56 (0.98) | 9.78 (0.43) |
|  | Accidental harm | 6.50 (2.07) | 7.28 (1.64) | 6.83 (2.31) | 7.11 (1.97) | 7.28 (1.49) | 6.83 (1.29) |
|  | Attempted harm | 6.72 (2.63) | 6.22 (2.73) | 8.83 (1.04) | 7.94 (2.10) | 7.56 (1.69) | 7.50 (1.89) |
|  | Nonharm | 1.78 (1.00) | 1.67 (0.97) | 3.17 (2.09) | 2.39 (1.85) | 2.28 (2.22) | 2.06 (1.83) |

**The Mean Reaction Time and SD across Conditions and Stimulation Types of the Pooled Sample**

| Condition | Time | R Anodal/L Cathodal | | L Anodal/R Cathodal | | Sham | |
| --- | --- | --- | --- | --- | --- | --- | --- |
|  |  | Mean | SD | Mean | SD | Mean | SD |
| Intentional harm | before | 9766.69 | 5764.74 | 17851.9 | 26267 | 13691.8 | 17055.1 |
|  | after | 7838.3 | 5689.6 | 11748.9 | 16724.7 | 12877 | 13608.4 |
| Accidental harm | before | 7263.9 | 4444.11 | 8764.22 | 7831.4 | 15821.5 | 33480.2 |
|  | after | 6013.16 | 3995.81 | 9316.84 | 14376.7 | 10348.6 | 10277.8 |
| Attempted harm | before | 5977.13 | 3454.66 | 13697.5 | 11633.5 | 10824.7 | 10821.2 |
|  | after | 5795.35 | 3095.81 | 11244.2 | 12070.6 | 9090.23 | 5692.02 |
| Nonharm | before | 9282.47 | 8118.39 | 13119.4 | 15168.9 | 13395.7 | 13555.1 |
|  | after | 7641.53 | 5681.83 | 10356.3 | 9012.82 | 9840.45 | 12968.2 |

**The Mean Reaction Time and SD across Conditions and Stimulation Types of Sample with Contexts**

| Context | Condition | Time | R Anodal/L Cathodal | | L Anodal/R Cathodal | | Sham | |
| --- | --- | --- | --- | --- | --- | --- | --- | --- |
|  |  |  | Mean | SD | Mean | SD | Mean | SD |
| economic interests | Intentional harm | before | 9986.74 | 6408.22 | 20363 | 26035.4 | 11886.2 | 15572.3 |
|  |  | after | 9626.48 | 6549.34 | 13269.4 | 20554.4 | 8207.52 | 10897.1 |
|  | Accidental harm | before | 7524.14 | 3570.35 | 9882.83 | 10160.3 | 20484.1 | 43950.8 |
|  |  | after | 5797.75 | 4276.74 | 8063.29 | 8029.52 | 12472.7 | 13430 |
|  | Attempted harm | before | 6344.09 | 3346.59 | 12129.7 | 8139.87 | 10355.1 | 10470.8 |
|  |  | after | 5900.15 | 3066.02 | 12180.4 | 15321.4 | 7930.52 | 3291.87 |
|  | Nonharm | before | 11997.2 | 10389.6 | 13653.6 | 13704.1 | 14796.8 | 14950.8 |
|  |  | after | 8465.74 | 6926.33 | 12381 | 9846.95 | 8748.86 | 9577.18 |
| relationships with friends | Intentional harm | before | 9546.65 | 5220.3 | 15340.9 | 27005.6 | 15497.4 | 18693.9 |
|  |  | after | 6050.11 | 4120.95 | 10228.3 | 12186.1 | 17546.5 | 14708.9 |
|  | Accidental harm | before | 7003.65 | 5269.83 | 7645.6 | 4515.2 | 11158.8 | 18168 |
|  |  | after | 6228.56 | 3805.71 | 10570.4 | 18913.8 | 8224.43 | 5249.82 |
|  | Attempted harm | before | 5610.18 | 3617.55 | 15265.3 | 14393.5 | 11294.3 | 11444.7 |
|  |  | after | 5690.55 | 3210.62 | 10308 | 7960.48 | 10249.9 | 7281.44 |
|  | Nonharm | before | 6567.75 | 3484.74 | 12585.1 | 16891.3 | 11994.6 | 12272.1 |
|  |  | after | 6817.32 | 4129.5 | 8331.62 | 7848.36 | 10932 | 15874.4 |
